# Supplementary material for: In-shoe plantar shear stress sensor design, calibration and evaluation for the diabetic foot
Source: PLoS One. 2024 Sep 4;19(9):e0309514. doi: 10.1371/journal.pone.0309514 (PMC11373826; doi:10.1371/journal.pone.0309514)
Supplement: Attachment — Submitted filename: Comments New.pdf [file pone.0309514.s001.pdf]

Manuscript title: In- Shoe Plantar Shear Stress Sensor Design, Calibration and Evaluation for the Diabetic Foot.

This work investigated the pressing issue of Diabetic Foot Ulceration (DFU), a significant global healthcare challenge. It highlights the crucial need for technology to identify the onset of DFU, enabling timely intervention, improving healthcare delivery, and reducing economic costs associated with its management. Specifically, the study focuses on the role of high-foot plantar stresses, including shear stress, in the formation of DFUs. It acknowledges the challenges in accurately measuring shear stress and the limited research in this area. It's commendable that the paper is well-written.

comments

- The abstract should emphasize the novelty of the research findings.
- The introduction section is crucial, but the author hasn't given it sufficient attention. It should include a discussion of currently available research.
- Abbreviations should be explained at the beginning. In the Methods section, there is confusion regarding the symbols used.
- The author should explain the effect of silicone stiffness on the strain gauge rosette.
- More detailed explanations of the experimental setup and testing methods are needed, including the number of subjects and their body parameters.
