# Supplementary material for: In-shoe plantar shear stress sensor design, calibration and evaluation for the diabetic foot
Source: PLoS One. 2024 Sep 4;19(9):e0309514. doi: 10.1371/journal.pone.0309514 (PMC11373826; doi:10.1371/journal.pone.0309514)
Supplement: Attachment — Submitted filename: Response to Reviewers.docx [file pone.0309514.s002.docx]

**Response to Reviewers**

We thank the reviewers for considering our manuscript and for providing their supportive feedback. We are pleased that the reviewers agree our work addresses the importance of creating a technology capable of measuring the full stress state of the diabetic foot to prevent ulceration and the accompanying challenges in measuring shear stress in a reliable and accurate manner.

Please see below for our response to the comments and suggestions made by the reviewers.

**Reviewer #1:**

**Reviewer Comment:** The Authors presented a work aimed at proposing the design and calibration of an in-shoe plantar shear stress sensor for applications in the assessment of diabetic foot ulceration. Specifically, focus is made on the evaluation in terms of sensor loading area and location. The manuscript is generally well written, but needs improvement. My major concern is about the description of the performed experiments in the methodology section. Indeed, the subdivision followed to describe calibration procedure and validation procedure is confusing thus not allowing the optimal fruition on what steps have been followed (e.g., the gait laboratory treadmill walking test is introduced at line 236 but participants are mentioned also in previous sections in the text). Providing an outline of the methodology content and/or improving subsections’ subtitles may be of help. Further minor comments are provided in the following.

**Authors response:** We thank you for your feedback and comments. We agree that due to the complex and intertwined analyses made in the study that the methodology and subdivisions could be made clearer and easier to follow. To help with this, we have added another figure in the paper to summarise the methodology and experiments that were made in the study, and added text to describe the experiments to follow as below:

**Manuscript Edit [page 5, line 75-78]:** This paper describes a sensor system design and conducts a performance investigation. Three investigations were conducted: calibration investigation, loading profile comparison and sensor validation. These investigations and how they relate to one another are shown in Fig 1.

**Fig 1. Summary of methodology for investigations into sensor calibration, loading profile and sensor validation.**

**Reviewer Comment:** - How were the compressive strains values for ε1 and ε2 determined? Please provide a brief sentence.

**Authors Response:** Thank you for your comment, the compressive strain regions were determined as the strain at the target low (14 kPa), medium (70 kPa) and high (140kPa) stresses from the stress-strain curve. This was obtained from the mechanical testing machine data (Instron) of the insole under normal (compressive) stress.

We have added the following in the manuscript for clarity:

**Manuscript Edit [page 5, line 88-92]:** The material properties (stress-strain relationship) for the silicone are non-linear but can be approximated as three linear regions (low: ${\leq\varepsilon}_{1}=0.04 strain$; medium: ${\leq\varepsilon}_{2}=0.115 strain$; high: ${>\varepsilon}_{2}strain)$; see Fig 2B. The strains for the three linear regions were determined from the stress-strain curve of the silicone under compressive loading at the target stresses of 14 kPa (low), 70 kPa (medium) and 140 kPa (high).

**Reviewer Comment:** In Fig. 3 label, “SSS” acronym is not defined. Same for “DAQ” in Fig. 4. Figures' labels should be self explaining.

**Authors Response:** We thank you for feedback and for highlighting these errors which we have amended in the manuscript as follows

**Manuscript Edit [page 8, Figure 3 caption]:** ‘Fig 3. Shear stress system (SSS) sensor and placement in the sensing insole…’

And moved lines 105-106 to the beginning of the section to explain the acronyms earlier in the text.

**Manuscript Edit [page 7, line 110-111]:** The shear stress sensing system primarily consists of the strain gauge rosette, a normal stress sensor, and the flexion stiffener and load concentrator; here on in referred to as the ‘shear stress system sensor’ or ‘SSS sensor’.

Authors Response: Similarly, we have made the following amendment for figure 4:

**Manuscript Edit [page 10, Figure 4 caption]:** Fig 4. Treadmill walking setup and data acquisition. [A] Participant walking on a treadmill with the sensor insole system. The data acquisition system (DAQ) was attached to a belt, and each insole (left and right foot) has a separate but identical DAQ input. [B] Block diagram of the DAQ system, collecting data at 80 Hz.

**Reviewer Comment:** Line 185: call to the figure is not clear.

**Authors Response:** We thank reviewer for their comment and for highlighting this error. We have amended as follows:

**Manuscript Edit [page 12, line 209-211]**: From the sensor calibration investigations in the results section, we can see that (i) loading location and (ii) loading area may affect the output of the SSS sensor so these must be considered during calibration.

**Reviewer Comment:** Line 205-207: the sentence is not clear, please rephrase. Some interrupted sentences are present in the text.

**Authors Response:** We thank reviewer for their comment. We have amended the sentences for better clarity as follows:

**Manuscript Edit [page 13, line 230-232]:** Comparisons were made between the participant’s mean normal stress profiles with the bench top test rig results (gait data averaged over 20 gait cycles from three different sensing locations hallux, first metatarsal head, and calcaneus, bench top test rig results for 15, 20, 30 and 40 mm indenter diameters).

**Reviewer Comment:** The reported state of art literature regarding the context in which the presented study may find application is very specific. I suggest the Authors to broaden the research context description by mentioning more in general where is the state of art for DFU risk prediction, in order to help the more general part of the Journal’s readership to become aware on the strength of the proposed work. Regarding to this, a recent review on “artificial intelligence methodologies applied to technologies for screening, diagnosis and care of the diabetic foot” may provide insightful information on which quantitative measures are suggested for an early identification of DFU risk, including plantar-based measurements. Indeed, in my opinion the quantitative information provided by shear stress measurement may also be used in artificial intelligence based approaches for the prediction of diabetic foot ulceration.

**Authors Response:** We thank the reviewer for their comment. We have amended the introduction section to include the wider context which may benefit from this type of shear stress measurement.

**Manuscript Edit [page 3, line 55-58]:**  Shear stress measurement is further complicated as all diabetic patients are strongly advised to walk using footwear (and never barefoot), therefore, to understand the shear stresses induced on the plantar surface, in-shoe shear stress measurement must be taken. Although direct shear stress measurement is important in DFU risk management, future use of artificial intelligence methods [13,14] may enable risk management with current measurement technologies.

**Reviewer #2:**

**Reviewer Comment:** This work investigated the pressing issue of Diabetic Foot Ulceration (DFU), a significant global healthcare challenge. It highlights the crucial need for technology to identify the onset of DFU, enabling timely intervention, improving healthcare delivery, and reducing economic costs associated with its management. Specifically, the study focuses on the role of high-foot plantar stresses, including shear stress, in the formation of DFUs. It acknowledges the challenges in accurately measuring shear stress and the limited research in this area. It's commendable that the paper is well-written.

**Authors Response:** We thank reviewer #2 for their comment

**Reviewer Comment:** The abstract should emphasize the novelty of the research findings.

**Authors Response:** We thank reviewer for their comment. We have amended the abstract to reflect more on the novelty of our research findings:

**Manuscript Edit [page 2, abstract]:** Plantar shear stress may have an important role in the formation of a Diabetic Foot Ulcer, but its measurement is regarded as challenging and has limited research. This paper highlights the importance of anatomical specific shear sensor calibration and presents a feasibility study of a novel shear sensing system which has measured in-shoe shear stress from gait activity on both healthy and diabetic subjects. The sensing insole was based on a strain gauge array embedded in a silicone insole backed with a commercial normal pressure sensor. Sensor calibration factors were investigated using a custom mechanical test rig with indenter to exert both normal and shear forces. Indenter size and location were varied to investigate the importance of both loading area and position on measurement accuracy. The sensing insole, coupled with the calibration procedure, was tested on one healthy and one diabetic participant during two sessions of 15 minutes of treadmill walking. Calibration with different indenter areas (from 78.5 mm^2^ to 707 mm^2^) and different positions (up to 40 mm from sensor centre) showed variation in measurements of up to 80% and 90% respectively. Shear sensing results demonstrated high repeatability (>97%) and good accuracy (mean absolute error < ±18 kPa) in bench top mechanical tests and less than 21% variability within walking of 15-minutes duration. The results indicate the importance of mechanical coupling between embedded shear sensors and insole materials. It also highlights the importance of using an appropriate calibration method to ensure accurate shear stress measurement. The novel shear stress measurement system presented in this paper, demonstrates a viable method to measure accurate and repeatable in-shoe shear stress using the calibration procedure described. The validation and calibration methods outlined in this paper could be utilised as a standardised approach for the research community to develop and validate similar measurement technologies.

**Reviewer Comment:** The introduction section is crucial, but the author hasn't given it sufficient attention. It should include a discussion of currently available research.

**Authors Response:** Thank you for your comment. We have included a discussion of the state of the art measurements of shear sensors as well as its wider application in the introduction section of the main text to reflect this.

**Manuscript Edit [page 3, line 44-54] :** Shear stress has been directly measured during barefoot gait using mechanical sensor arrays coupled with resistive or capacitive sensors [6-8], utilising piezoelectric materials and their charge outputs [9] and through a variety of optical methods including polycarbonate arrays [6], optical bend loss [7] and laser interferometry of bi-refringent films [8]. Perry et al [10] used an array-based device [11] to study bunching and stretching of adjacent plantar tissue and they found that tissue stretching from shear stress was the predominant mechanism. They report that peak shear stress and peak plantar pressure occur in the same place in 50% of cases, but actually occur at different times, which is contradictory to results reported by other researchers [12]. Contradictory results are typical from these studies using custom-built shear stress measurement devices due to the relatively low numbers of diabetic patients tested in the trials, with typical sample sizes of ten. All these measurement methods are bespoke devices and only a handful of foot-to-floor shear stress measurement devices exist worldwide. Larger scale studies with matched control groups are required to provide firm conclusions on plantar surface shear stresses experienced by diabetics.

Shear stress measurement is further complicated as all diabetic patients are strongly advised to walk using footwear (and never barefoot), therefore, to understand the shear stresses induced on the plantar surface, in-shoe shear stress measurement must be taken. Although direct shear stress measurement is important in DFU risk management, future use of artificial intelligence methods [13,14] may enable risk management with current measurement technologies.

**Reviewer Comment:** Abbreviations should be explained at the beginning. In the Methods section, there is confusion regarding the symbols used.

**Authors Response:** Thank you for your feedback. We have amended the method sections to ensure each abbreviation is explained in full upon first appearance in the text. Please see the following amendments

**Manuscript Edit [page 8, Figure 3 caption]:** ‘Fig 3. Shear stress system (SSS) sensor and placement in the sensing insole…’

And moved lines 105-106 to the beginning of the section to explain the acronyms earlier in the text.

**Manuscript Edit [page 7, line 110-111]:** The shear stress sensing system primarily consists of the strain gauge rosette, a normal stress sensor, and the flexion stiffener and load concentrator; here on in referred to as the ‘shear stress system sensor’ or ‘SSS sensor’.

Authors Response: Similarly, we have made the following amendment for figure 4:

**Manuscript Edit [page 10, Figure 4 caption]:** Fig 4. Treadmill walking setup and data acquisition. [A] Participant walking on a treadmill with the sensor insole system. The data acquisition system (DAQ) was attached to a belt, and each insole (left and right foot) has a separate but identical DAQ input. [B] Block diagram of the DAQ system, collecting data at 80 Hz.

**Reviewer Comment:** The author should explain the effect of silicone stiffness on the strain gauge rosette.

**Authors Response:** Thank you for your comment. We agree that this can be discussed in further detail. The choice of silicone stiffness was important for two reasons: (i) the strain limitations of the gauge, and the (ii) resulting sensitivity of the assembled sensor. The stiffness of the silicone cannot be too low that the stresses will cause the sensor to exceed the strain limit of the gauge and break the gauge, and cannot be too high that it does not provide the required sensitivity to measure the minimum stresses. The strain gauge with the highest strain limit available was chosen to give way for a less stiff silicone to be chosen. However, both the choice of strain gauge and silicone stiffness was limited to the available resources and commercially available options. As our choice of silicone and strain gauge allowed us to create a sensor that had the range and sensitivity within our requirements, we did not explore this further. Future work could investigate these effects in a more systematic manner.

We have added a brief sentence in the limitations section of the manuscript to explain this effect and that future investigations are needed.

**Manuscript Edit [page 28, line 449-451]** : Relative stiffness of the silicone and the strain gauge rosette will affect strain transfer between the two materials. Material properties of the silicone is highly important for measurement accuracy, sensitivity, and range, and warrants further investigation.

**Reviewer Comment:** More detailed explanations of the experimental setup and testing methods are needed, including the number of subjects and their body parameters.

**Authors Response:** Thank you for your feedback. We agree that that this information may be missed due to the multiple sections and studies made in this paper. We have amended the manuscript structure to provide better clarity by adding a figure in the methodology section to describe the investigations in the study.

**Manuscript Edit [page 5, line 75-77]:** This paper describes a sensor system design and conducts a performance investigation. Three investigations were conducted: calibration investigation, loading profile comparison and sensor validation. These investigations and how they relate to one another are shown in Fig 1 (see earlier response to reviewer 1)
