# Supplementary material for: In-shoe plantar shear stress sensor design, calibration and evaluation for the diabetic foot
Source: PLoS One. 2024 Sep 4;19(9):e0309514. doi: 10.1371/journal.pone.0309514 (PMC11373826; doi:10.1371/journal.pone.0309514)
Supplement: Attachment — Submitted filename: Response to Reviewers July 2024.docx [file pone.0309514.s003.docx]

Response to Reviewers 17-07-2024

**Authors’ General Response:**

We thank the all reviewers for their thorough and constructive review of our paper, and are pleased to attach the following minor amendments to the paper as suggested

**Reviewer 1 Comment #1:** The Authors responded to almost all Reviewers' comments, but information on anthropometric characteristics and body parameters is not yet reported, thus compromising reproducibility.

**Author’s Response**: Thank you for your comment, we appreciate that this is important information for reproducibility.

We have stated the following anthropomorphic parameters at the start of the ‘sensor validation section’ as follows: ‘*To further validate the sensors, a gait laboratory treadmill walking test was performed on a single anthropometrically matched healthy participant and a single diabetic participant (both male and 45 years old, weighing 88 kg and 75 kg, EU shoe size 44 and 42, weight per insole area 32 kPa and 35 kPa, walking speed 0.92 ms^-1^ and 0.95 ms^-1^ for the healthy and diabetic respectively)…*’. These parameters were disclosed as they were similar parameters described in the other similar study in PLOS ONE of measuring in-shoe shear (Tang et. al, 2023). We have added *height* details to match this paper as follows:

**Manuscript Edit (page 15, line 262-265): ‘**To further validate the sensors, a gait laboratory treadmill walking test was performed on a single anthropometrically matched healthy participant and a single diabetic participant (both male and 45 years old, weighing 88 kg and 75 kg, height of 1.75 and 1.66 m, EU shoe size 44 and 42, weight per insole area 32 kPa and 35 kPa, walking speed 0.92 ms^-1^ and 0.95 ms^-1^ for the healthy participant and participant with diabetes respectively)

**Reviewer 1 Comment #2:** Furthermore, the authors should prefer to use “people with diabetes” or similar, instead of “diabetics”.

**Author’s Response:** Thank you for your comment, we agree that this is preferable. As such, we’ve changed this on the following sections/sentences in the manuscript (italicized):

**Abstract Edit: ‘…**The sensing insole, coupled with the calibration procedure, was tested one *participant with diabetes* and one healthy participant during two sessions of 15 minutes of treadmill walking. Calibration with different indenter areas (from 78.5 mm^2^ to 707 mm^2^) and different positions…’

**Manuscript Edit (page 3, line 41): ‘**Diabetic foot ulceration (DFU) affects 15-25% of *people with diabetes* at some point in their lifetime [1]’

**Manuscript Edit (page 3, line 52-53): ‘**Contradictory results are typical from these studies using custom-built shear stress measurement devices due to the relatively low numbers of *participants with diabetes tested* in the trials, …’

**Manuscript Edit (page 3, line 55-56):** ‘Larger scale studies with matched control groups are required to provide firm conclusions on plantar surface shear stresses experienced by *people with diabetes*.’

**Manuscript Edit (page 12, line 264-267): ‘**To further validate the sensors, a gait laboratory treadmill walking test was performed on a single anthropometrically matched healthy participant and a single diabetic participant (both male and 45 years old, weighing 88 kg and 75 kg, height of 1.75 and 1.66 m, EU shoe size 44 and 42, weight per insole area 32 kPa and 35 kPa, walking speed 0.92 ms^-1^ and 0.95 ms^-1^ for the healthy participant and participant with diabetes respectively).

**Manuscript Edit** **(page 13, line 279-280):** ‘…designed with shock-absorbing properties and a larger volume, ideal for *people with diabetes.’*

**Manuscript Edit** **(page 19, line 402-403)**: ‘Loads were cyclic going from zero to peak value with the same frequency as gait which were at speeds of 0.92 and 0.95ms^-1^ for the healthy participant and **participant with diabetes** respectively.’

**Manuscript Edit** **(page 20, column 1,** **Table 4**)

**Manuscript Edit** **(page 21, line 410-412)**: ‘However, this study aimed to demonstrate the feasibility, accuracy, and repeatability of the SSS system so no conclusions should be drawn on plantar stress for general *people with diabetes* and healthy populations for this study.’

**Manuscript Edit** **(page 21, line 415-420):** ‘Intra-walk differences were lower than inter-walk – with the highest percentage difference of 21% measured by the SSS sensor for the ML Shear (Hallux, Left foot, *participant with diabetes*). Other measurements from the shear stress sensors were < 15% difference. For inter-walk, the highest PPS percentage difference was measured by the commercial Flexiforce sensor of 47% difference in normal stress (Hallux, left foot, *participant with diabetes*), followed by 37% for the AP shear of the SSS sensor (Hallux, right, healthy) and 33% for the ML shear of the SSS sensor (Calcaneus, right, *participant with diabetes*).’

**Manuscript Edit** **(page 23 line 462-464):** ‘..Despite observing minimal shear sensor temperature response, variability between 20–30 °C, literature indicates foot temperatures may be as high as 35° in people with diabetes [39, 40]..’

**Reviewer 1 Comment #3:** Even if the effort made to improve the explanation of the methodology is recognisable, Figure 1 should still be referred to in the text wherever necessary (perhaps adding letters to the panels could help this purpose). The resolution of Figure 1 should also be improved.

**Author’s Response**: Thank you for your comment, we have included, where necessary, references to Figure 1 in the main manuscript as suggested and added letters to panels as well as improved the quality of the figure.

**Manuscript Edit** **(page 8 line 172-173):** To investigate the effect of calibration on the sensor’s performance, both shear and normal force were applied to the SSS sensor insole (summarised in Fig 1A).

**Manuscript Edit** **(page 11 line 231-232):** An illustrated summary of this investigation can also be found in Fig 1B.

**Manuscript Edit** **(page 14 line 244):** The following section describes the sensor validation, as summarised in Fig 1C.

**Reviewer 2 comment:** The authors have thoroughly addressed all the comments, demonstrating a positive and constructive approach. They have provided detailed responses and made the necessary revisions to improve the manuscript, ensuring that all concerns and suggestions from the reviewers have been adequately considered and integrated.

**Authors’ Response:** Thank you for reviewing our study and providing helpful feedback to improve our paper, we greatly appreciate this. We note from your comment that you are satisfied with our edits in response to your feedback.

**Other amendments:**

After a resubmission review of the manuscript, we have also identified a necessary amendment and have made this edit in the manuscript as follows (italicised):

**Manuscript Edit (page 15, line 326-329):** ‘Other researchers measured in-shoe peak shear stresses from gait varied from 9 kPa to 140 kPa and calibration loading area varied from 20 mm diameter area (314 mm^2^) –*10,000 mm^2^ (up to half the insole, approximated from the experimental figure 3 in the paper as there was insufficient detail to give conclusive information on the loading area used)* [5,15].’
